# Transcriptomic Profiling of Diabetic Porcine Wound Healing Model Identifies Key Metabolic, Inflammatory, and Oxidative Stress Pathways

**DOI:** 10.1111/wrr.70185

**Published:** 2026-07-02

**Authors:** Joshua T. McCune, Mariah G. Bezold, Jeffrey M. Davidson, C. Henrique Serezani, Rebecca S. Cook, Craig L. Duvall

**Affiliations:** ^1^ Department of Biomedical Engineering Vanderbilt University Nashville Tennessee USA; ^2^ Department of Pathology, Microbiology and Immunology Vanderbilt University School of Medicine Nashville Tennessee USA; ^3^ Department of Medicine, Division of Infectious Diseases Vanderbilt University Medical Center Nashville Tennessee USA

**Keywords:** diabetic foot ulcer, porcine model, RNA‐seq, streptozotocin, wound healing

## Abstract

Diabetic foot ulcers remain a major clinical challenge as diabetes prevalence rises, emphasising the need for improved therapeutics and relevant preclinical models. Common rodent wound‐healing models poorly recapitulate human skin anatomy and repair. Although porcine skin is comparable to human skin, many studies employ young, healthy pigs that do not reflect typical diabetic human wounds. Here, we evaluated wound healing in full‐thickness skin wounds in non‐diabetic and diabetic Yucatan minipigs. RNA sequencing identified key transcriptional differences in wounds of diabetic versus non‐diabetic animals, including pathways linked to increased inflammation and oxidative stress, as well as decreased metabolism and extracellular matrix organisation, known hallmarks of diabetic wounds. These findings support this preclinical model as a powerful approach for discovery and therapeutic testing in diabetic wounds and provide a novel data set for further mining of potential gene targets for diabetic wound intervention.

## 1 Introduction

Diabetic foot ulcers (DFUs) are associated with high rates of lower limb amputation and increased patient mortality [1]. The DFU microenvironment is complex—involving persistent inflammation, dysregulation of fibroblasts and keratinocytes, imbalanced extracellular matrix (ECM) production, oxidative stress, and metabolic dysfunction, all of which delay wound healing [2]. With an aging population and rising diabetes rates, there is a pressing need to improve DFU therapies. However, testing of new treatments and drugs is limited to a few suitable animal models that recapitulate the diabetic condition and molecular factors driving DFUs. Although commonly used diabetic mouse wound‐healing models (e.g., streptozotocin‐induced, db/db, NOD) have provided valuable insight, they lack key features of human skin and heal primarily through contraction, necessitating stenting for accurate wound‐healing studies [2]. Porcine wound‐healing models are more relevant due to their anatomical and physiological similarities to human skin; however, comprehensive characterisation of diabetic porcine models remains limited [2, 3, 4]. In this study, we have characterised a streptozotocin‐induced (STZ) porcine model of full‐thickness wound healing at an early time point, detailing evidence of delayed wound closure and, for the first time, comparing bulk transcriptomic differences across diabetic and non‐diabetic wounds within this model.

## 2 Materials and Methods

All materials and methods are detailed in the Supporting Information Materials and Methods section.

## 3 Results and Discussion

### 3.1 Streptozotocin‐Induced Diabetes Impairs Wound Healing in Yucatan Minipigs

**FIGURE 1:**
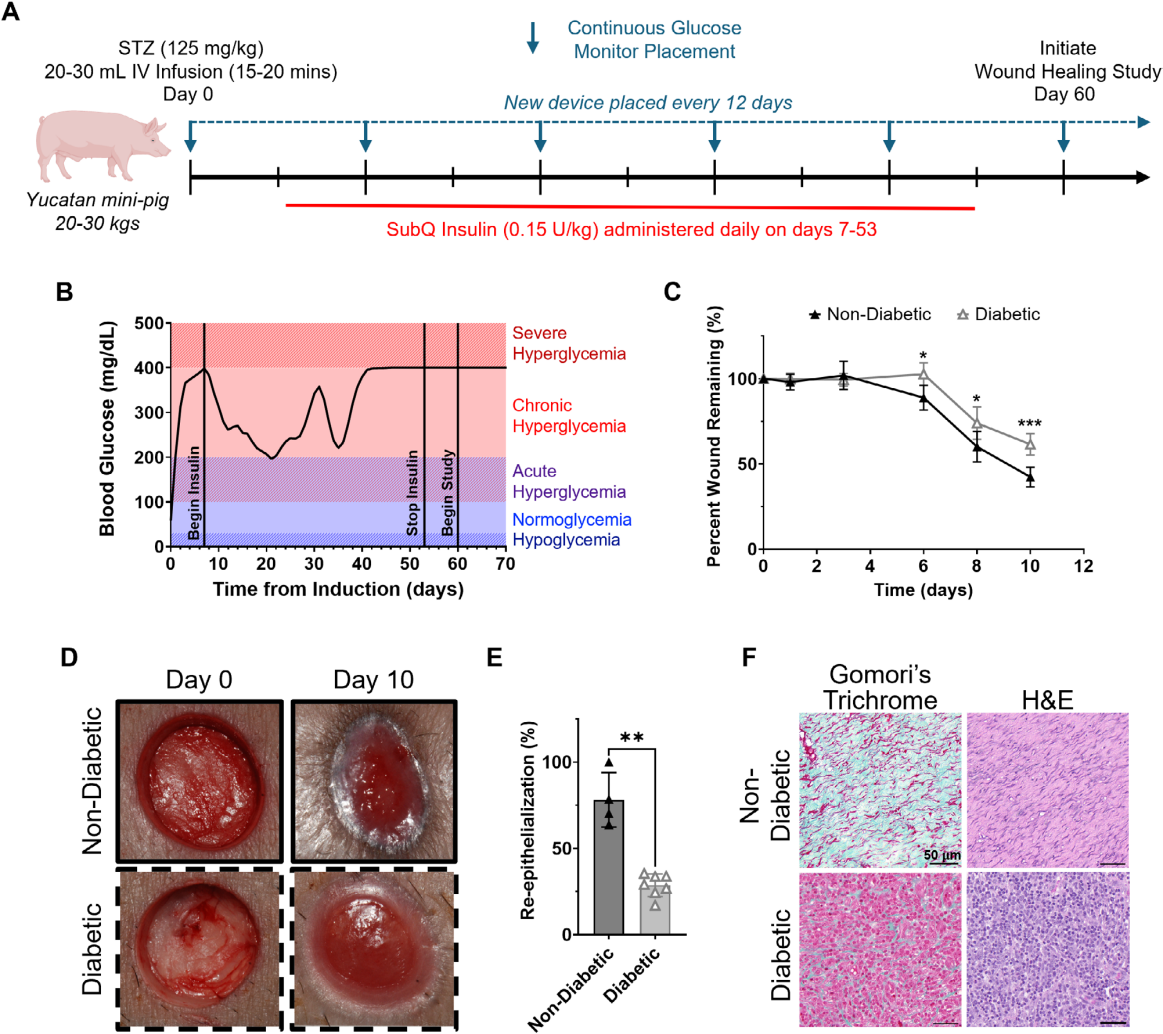
Streptozotocin induction and wound healing study in Yucatan minipig. (A) Scheme and timeline of STZ induction and wound healing study, (B) daily average blood glucose concentration of diabetic minipig, (C) full‐thickness wound closure rates over time quantified from macroscopic photographs of the wounds (on‐Diabetic *N* = 4, Diabetic *N* = 7), (D) representative images of wounds at day 0 and 10, (E) re‐epithelialization of wounds at day 10 quantified from immunohistochemistry (IHC) of cytokeratin14, and (F) representative histology of wounds at day 10. *p < 0.05, **p < 0.01, and ***p < 0.001, by Welch's unpaired t‐test.

**FIGURE 2:**
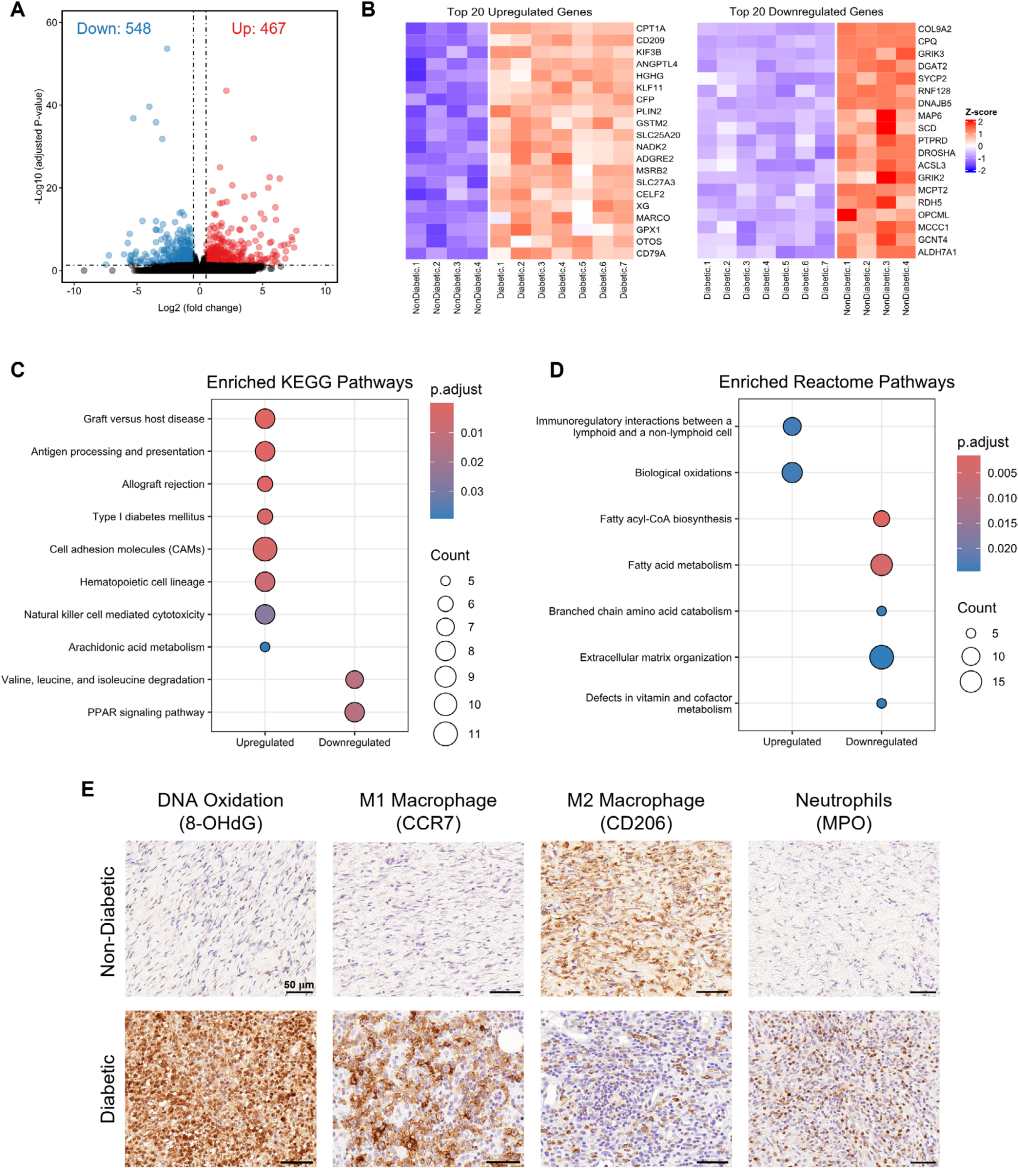
Differential gene expression analysis, functional enrichment analysis, and immunohistochemistry (IHC) comparing non‐diabetic (*N* = 4) and diabetic wounds (*N* = 7). (A) Volcano plot of differentially expressed genes, (B) heatmaps of top 20 upregulated and downregulated genes within the gene set, (C) KEGG enrichment analysis results, (D) Reactome enrichment analysis results, and (E) representative IHC of immune cell populations in diabetic and non‐diabetic wounds on day 10.

We induced diabetes in a Yucatan minipig via intravenous infusion of STZ (125 mg/kg), followed by a 60‐day maturation period with supplemental insulin to establish a hyperglycemic state while maintaining animal viability (Figure 1A). The diabetic state was confirmed based on the classic symptoms of hyperglycemia (e.g., polyuria, polydipsia) and a blood glucose level in the hyperglycemic range (≥ 200 mg/dL) (Figures 1B, S1A,B) [5]. Insulin was discontinued one week before wounding to mirror clinical scenarios in which diabetes remains poorly controlled and promote the underlying pathophysiology of chronic wounds. Full‐thickness skin wounds were created 60 days after diabetes induction using a 2 cm biopsy punch. Identical wounds were generated in a non‐diabetic Yucatan minipig to compare the effect of diabetes on wound healing in this model. Wound area was measured over 10 days, after which the study was terminated, capturing an early wound‐healing timepoint to assess differences in the resolution of inflammation, granulation tissue formation, and re‐epithelialization. The diabetic wounds demonstrated significant delays in wound closure and contraction, quantified by digital planimetry (Figures 1C,D, S1C), as well as epithelial migration/re‐epithelialization, quantified by immunostaining for cytokeratin 14 (Figures 1E, S1D), compared with non‐diabetic wounds. Histological examination of diabetic wounds revealed striking differences in immune cell infiltration and phenotype with reduced collagen/granulation tissue deposition compared with non‐diabetic wounds (Figures 1F, 2F and S2). These results highlight diabetes‐associated wound‐healing impairments, including delayed wound closure, diminished matrix deposition, and decreased re‐epithelialization, closely resembling the wound healing deficits that characterise human DFUs [6, 7, 8].

### 3.2 Wounds in Diabetic Minipigs Exhibit Different Transcriptional Profiles Than Non‐Diabetic Wounds

Bulk RNA sequencing of diabetic wounds (*N* = 7) revealed distinct gene expression profiles compared to non‐diabetic wounds (*N* = 4), with 1015 genes demonstrating differential expression (|Log2FC| > 0.5, adjusted *p* < 0.05) (Figure 2A). The 20 most activated and suppressed genes (Figure 2B) include those related to metabolic dysfunction (CPT1A, PLIN2, SLC25A20, NADK2, SLC27A3, CPQ, DGAT2, SCD, PTPRD, ACSL3, MCCC1, ALDH7A1), oxidative stress (GST, MSRB2, GPX1), and inflammation (CD209, IGHG, CFP, CELF2, MARCO, CD79A, RNF128). Functional enrichment analysis further corroborated these molecular signatures, revealing four major categories of dysregulated pathways (Figures 2B,C, S3–S16).

### 3.3 Inflammation and Immune Dysregulation Characterise Wounds of Diabetic Minipig

Upregulated genes related to innate and adaptive immunity were enriched in autoimmune‐associated KEGG pathways, consistent with previous reports of STZ‐induced diabetes in rodents (graft versus host disease, allograft rejection, and Type I diabetes mellitus) [9, 10, 11]. Critical genes enriched in these pathways include TNF and major histocompatibility (MHC) complex genes (SLA‐DOB, SLA‐DMA, SLA‐8, SLA‐DRA). This enrichment aligns with the mechanism of STZ‐induced autoimmunity, which triggers MHC upregulation and the generation of auto‐reactive lymphocytes [12]. These upregulated genes also implicate activated antigen presentation and immune surveillance within diabetic wounds, consistent with hyperglycemia‐induced changes in macrophage‐directed immunity [13, 14].

Genes encoding cell adhesion molecules (CAMs) involved in immune cell extravasation and infiltration into tissue (LTCAM, ITGAM, SELL, ICAM3) were significantly upregulated in diabetic wounds. Elevated levels of immune cell infiltration were also detected through increased CCR7 and myeloperoxidase (MPO) immunostaining, reflecting increased M1 pro‐inflammatory macrophage and neutrophil infiltration, respectively, in diabetic wounds (Figure 2E). Similarly, haematopoietic cell lineage pathways were enriched in diabetic wounds, indicating local immune cell expansion (FCER2, CSF2RA, CD14, ITGAM, CD22, SLA‐DRA, TNF, and IL1R2). Reactome enrichment analysis further suggested that diabetic wounds harboured excessive, pathological pro‐inflammatory immune activation. Transcriptomic conclusions were corroborated by the elevated cellularity and immune infiltration observed histologically. The natural killer (NK) cell‐mediated cytotoxicity pathway was also enriched. Strikingly, enriched genes represented both activating and inhibitory NK receptors (TNF, FCGR3A, SLA‐8, PIK3R5, SHC2, KLRD1, RAC2, and KLRC1). These findings are consistent with observed NK cell dysregulation in diabetes, contributing to oxidative stress and inflammatory tissue damage [15, 16].

### 3.4 Oxidative Stress Is Upregulated in Wounds of Diabetic Minipig

Enrichment in arachidonic acid metabolism and biological oxidation pathways, with upregulation of oxidative response genes (PTGES, PTGS1, CYP2B6B, GPX1, and GGT5) is consistent with elevated ROS burden in diabetic wounds. This hypothesis was confirmed by 8‐OHdG immunostaining, reflecting increased DNA oxidative damage in diabetic wounds (Figures 2E, S2A). Elevated ROS in diabetic wounds has been noted previously and is multifactorial, but hyperglycemia is known to activate NAPDPH oxidases, impair antioxidant enzyme activity, and drive mitochondrial dysfunction [8, 17]. Additionally, oxidative stress in DFUs impacts the function of key cell types, predominantly endothelial cells, keratinocytes, and fibroblasts, ultimately impairing proliferation, migration, and differentiation [7].

### 3.5 Metabolic Dysfunction and Energy Depletion in Wounds of Diabetic Minipig

Diabetic wounds also demonstrated downregulation of genes involved in various metabolic pathways, including branched chain amino acid (BCAA) catabolism (IVD, 1CD2097A1, OXCT1, MMUT, and ACADSB). This reduction causes BCAA accumulation and increases production of their ketoacid byproducts (BCAKs), thus exacerbating insulin resistance, mitochondrial dysfunction, and ROS generation, while also impairing ATP generation essential for cell proliferation, migration, and collagen synthesis [18, 19].

Similarly, downregulation of key genes associated with fatty acid (FA) metabolism (ELOVL5, THRSP, ACLY, ACSL1, SCD, ELOVL6, PON3, ACACA, CYP1A1, CYP4B1, MORC2, CYP2U1, FADS1, ACSL3, MMUT) and peroxisome proliferator‐activated receptor (PPAR) signalling (OLR1, ME1, ACSL1, SCD, SLC27A6, FABP3, LPL, and ACSL3) indicates impaired lipogenic capacity and energy substrate utilisation in diabetic wounds. Given that FA oxidation (FAO) is critical for efficient ATP production, reduced expression of FAO‐related genes likely compromises the energetic capacity of cells in diabetic wounds [19]. FAO is critical for efficient oxidative phosphorylation and is therefore a key factor in the phenotypic transition of macrophages from pro‐inflammatory M1, a phenotype reliant on glycolysis, to pro‐resolution M2, which requires oxidative phosphorylation [14, 19]. Chronic downregulation of FAO‐related genes may prevent M1‐to‐M2 macrophage polarisation. Indeed, CCR7 immunostaining revealed increased pro‐inflammatory (CCR7+) macrophage accumulation, while pro‐resolution (CD206+) macrophages were substantially decreased in diabetic wounds compared to non‐diabetic wounds (Figures 2E, S2B,C), consistent with impaired M1‐to‐M2 macrophage polarisation, which is known to occur in hyperglycemic environments and is characteristic of dysregulated inflammation in human DFUs [20]. Importantly, M1 macrophages perpetuate pro‐inflammatory cytokine production (e.g., TNF) and ROS generation, thereby sustaining inflammation and oxidative stress [14, 19].

These gene expression changes are consistent with essential roles of BCAA and FA metabolism in wound healing and inflammation, identifying potential molecular targets for further exploration.

### 3.6 ECM Organisation Dysfunction in Wounds of Diabetic Minipigs

Genes associated with ECM production and organisation were robustly downregulated, including collagen isoforms (COL8A1, COL9A2, COL15A1, COL25A1), crosslinking enzymes (COLGALT2, P4HA1, PLOD2), matricellular proteins (TGFB3, FBN1, SPP1), and mediators of cell‐ECM interactions (LAMA1, LAMA3, ACAN, ITGA8). Downregulation of these components may result in an insufficient, immature, poorly cross‐linked matrix, adversely affecting cell migration and tissue integrity. Diminished and disorganised ECM has been noted previously in human DFUs and is thought to affect cell‐matrix signalling required for fibroblast and keratinocyte migration and differentiation [7, 19, 21]. These transcriptomic results align with the histological observations of reduced collagen deposition and altered tissue architecture in the diabetic wounds (Figure 1F).

Single‐cell and bulk sequencing studies of diabetic wounds in humans and rodents corroborate the molecular signatures and dysregulated pathways observed in these porcine diabetic wounds. Comparisons of non‐diabetic and diabetic wounds have demonstrated elevated levels of pro‐inflammatory immune cells, predominantly monocytes and macrophages, in diabetic wounds, and upregulated inflammatory response pathways, thereby perpetuating a dysregulated immune milieu in DFUs [6, 20, 22]. Additionally, recent work in both diabetic humans and mice has identified oxidative stress pathways and metabolic reprogramming shifting toward a glycolysis‐dependent phenotype, enriched in fibroblast/stromal cells, negatively impacting their proliferation, migration, and collagen synthesis, contributing to disorganised ECM deposition and impaired wound healing [22, 23, 24]. These fibroblast/stromal cells also exhibit increased expression of inflammatory mediators, further perpetuating the dysregulated immune environment that contributes to impaired wound healing [23, 25]. Together, these key pathways represent the complex diabetic wound environment characterised by dysregulated inflammation, oxidative stress, metabolic reprogramming, and impaired ECM deposition.

## 4 Conclusion

In the present work, we transcriptionally profiled a preclinical diabetic wound‐healing model in Yucatan minipigs. Induction of diabetes by STZ resulted in diabetic wounds that provided evidence of delayed rates of wound closure compared to non‐diabetic wounds. Transcriptomic analysis identified enriched autoimmune pathways, elevated oxidative stress, dysregulated metabolic pathways, and comprehensive suppression of ECM organisation. These findings were supported by histological findings of increased immune cell infiltration and decreased ECM deposition in diabetic wounds. Our findings confirm that many of the pathophysiological and molecular hallmarks of human DFUs are recapitulated in this diabetic porcine model. Ultimately, anatomical similarity between pig and human skin and robust generation of diabetic wound pathology position this model as a valuable preclinical platform for testing emerging therapeutics in a physiologically relevant context. Together, the gene signatures identified here point to tractable pathways that may contribute to pathological wound healing in diabetic patients, providing a resource to be further mined for new therapeutic targets.

## 5 Limitations

We acknowledge there are several limitations of the work presented. First, these data provide only a static view of the wound healing response at a single time point. Longitudinal studies could help to fully characterise the dynamics of the wound‐healing response by evaluating the time to complete wound closure and transcriptional shifts throughout the healing process in this model. Secondly, while the use of bulk sequencing enables holistic analysis of transcriptional profiles in wound tissue samples, it lacks the resolution to capture the phenotype and expression profile of individual cell types. Single‐cell sequencing or spatial transcriptomics could provide a more robust understanding of the pathobiology of porcine diabetic wounds. Lastly, while this model successfully demonstrates key features of healing diabetic wounds, the wounds do ultimately heal; as such, they may not reflect true chronic non‐healing wounds, but rather impaired acute wounds.

## Funding

This work was supported by the National Institutes of Health (NIH) (R01 EB02869, R01 EB019409, T32 DK101003, and P30 CA068485).

## Conflicts of Interest

The authors declare no conflicts of interest.

## Supporting information


**Table S1:** Differentially expressed genes in diabetic porcine wounds compared to non‐diabetic wounds, defined as having a |Log2FC| ≥ 0.5 and adjusted *p*‐value < 0.05.

**Table S2:** Enriched KEGG and Reactome pathways, and their associated differentially expressed genes, in diabetic porcine wounds compared to non‐diabetic wounds.

**Figure S1:** Streptozotocin induction and wound healing study in Yucatan minipig. (A) Average (mean ± std.) temporal daily blood glucose (mg/dL) and (B) body weight of diabetic minipig over the course of the study. (C) Representative images of wounds at day 0, 1, 3, 6, 8, and 10. (D) Representative IHC of re‐epithelialization by cytokeratin 14 staining in diabetic and non‐diabetic wounds at day 10, with dashed lines and arrows indicating the edge of the epithelial tongue.
**Figure S2:** Quantification of (A) 8‐OHdG, (B) CCR7, and (C) CD206 immunohistochemistry as a percentage of positive cells in the wound bed. *p < 0.05, **p < 0.01, and ***p < 0.001, by Welch's unpaired t‐test.
**Figure S3:** Differential gene expression and functional enrichment analysis. (A) Clustered heatmap of differentially expressed genes (DEGs), with row cluster 1 corresponding to upregulated genes in diabetic wounds and row cluster 2 corresponding to downregulated genes. (B) Gene ontology biological processes enriched within diabetic wounds.
**Figure S4:** KEGG enrichment—Graft versus host disease. (A) Pathway schematic illustrating genes that were upregulated (red) within the pathway in diabetic vs. non‐diabetic wounds. (B) Clustered heatmap of upregulated genes within the graft versus host disease pathway.
**Figure S5:** KEGG enrichment—Antigen processing and presentation. (A) Pathway schematic illustrating genes that were upregulated (red) within the pathway in diabetic vs. non‐diabetic wounds. (B) Clustered heatmap of upregulated genes within the antigen processing and presentation pathway.
**Figure S6:** KEGG enrichment—Allograft rejection. (A) Pathway schematic illustrating genes that were upregulated (red) within the pathway in diabetic vs. non‐diabetic wounds. (B) Clustered heatmap of upregulated genes within the allograft rejection pathway.
**Figure S7:** KEGG enrichment—Type I diabetes mellitus. (A) Pathway schematic illustrating genes that were upregulated (red) and downregulated (green) within the pathway in diabetic vs. nondiabetic wounds. (B) Clustered heatmap of upregulated genes within the type I diabetes mellitus pathway.
**Figure S8:** KEGG enrichment—Cell adhesion molecules (CAM) interactions. (A) Pathway schematic illustrating genes that were upregulated (red) and downregulated (green) within the pathway in diabetic vs. nondiabetic wounds. (B) Clustered heatmap of upregulated genes within the cell adhesion molecules (CAM) interactions pathway.
**Figure S9:** KEGG enrichment—Haematopoietic cell lineage. (A) Pathway schematic illustrating genes that were upregulated (red) and downregulated (green) within the pathway in diabetic vs. nondiabetic wounds. (B) Clustered heatmap of upregulated genes within the haematopoietic cell lineage pathway.
**Figure S10:** KEGG enrichment—Natural killer cell mediated cytotoxicity. (A) Pathway schematic illustrating genes that were upregulated (red) within the pathway in diabetic vs. nondiabetic wounds. (B) Clustered heatmap of upregulated genes within the natural killer cell mediated cytotoxicity pathway.
**Figure S11:** KEGG enrichment—Arachidonic acid metabolism. (A) Pathway schematic illustrating genes that were upregulated (red) and downregulated (green) within the pathway in diabetic vs. nondiabetic wounds. (B) Clustered heatmap of upregulated genes within the arachidonic acid metabolism pathway.
**Figure S12:** KEGG enrichment—Valine, leucine, and isoleucine degradation. (A) Pathway schematic illustrating genes that were downregulated (green) within the pathway in diabetic vs. nondiabetic wounds. (B) Clustered heatmap of downregulated genes within the Valine, leucine, and isoleucine degradation pathway.
**Figure S13:** KEGG enrichment—PPAR signalling pathway. (A) Pathway schematic illustrating genes that were upregulated (red) and downregulated (green) within the pathway in diabetic vs. nondiabetic wounds. (B) Clustered heatmap of downregulated genes within the PPAR signalling pathway.
**Figure S14:** Upregulated Reactome enrichment. (A) Clustered heatmap of genes upregulated within the immunoregulatory interactions between lymphoid and non‐lymphoid cell pathway in diabetic relative to nondiabetic wounds. (B) Clustered heatmap of genes upregulated within the biological oxidations pathway in diabetic relative to nondiabetic wounds.
**Figure S15:** Downregulated Reactome enrichment—Clustered heatmap of genes downregulated in diabetic relative to nondiabetic wounds within the (A) fatty acyl‐CoA biosynthesis, (B) branched chain amino acid catabolism, (C) fatty acid metabolism, and (D) defects in vitamin and cofactor metabolism pathways.
**Figure S16:** Downregulated Reactome enrichment—Clustered heatmap of genes downregulated in diabetic relative to nondiabetic wounds within the extracellular matrix organisation pathway.

## Data Availability

All data generated in this study are available within the article or Supporting Information or from the corresponding author upon reasonable request. Gene expression data generated and analysed in this study are available in the Gene Expression Omnibus (GEO) repository, accession number GSE315429.
